# Supplementary material for: Different Oncologic Outcomes in Early-Onset and Late-Onset Sporadic Colorectal Cancer: A Regression Analysis on 2073 Patients
Source: Cancers (Basel). 2022 Dec 18;14(24):6239. doi: 10.3390/cancers14246239 (PMC9777335; doi:10.3390/cancers14246239)
Supplement: Supplementary file 1 [file cancers-14-06239-s001.zip › cancers-2033140-supplementary.pdf]

Table S1. Detailed list of the variables included in the prospective institutional database and the variables selected to be analyzed in the study

Variable category	Prospective institutional database	Selected variables
Demographic	Date of birth; Place of birth; Gender; Date of diagnosis; Age at diagnosis.	Gender; Age at diagnosis
Clinical information	Weight; Height; BMI; Previous medical history; CCI; Previous and concomitant medications; Previous surgeries; Smoking status; Alcohol addiction; Relevant infections; Tumor location; Family history of CRC (and degree relatives); Family history of tumor (and degree relatives); Known history of genetic syndromes; Type of genetic syndrome.	BMI; Previous medical history; CCI; Smoking status; Tumor location; Family history of CRC (and degree relatives); Family history of tumor (and degree relatives); Known history of genetic syndromes (if yes, patients were excluded).
Therapeutics information	Preoperative chemo or chemoradio treatment; Hospital of treatment; Start date and end date of treatment; Type of treatment; Indication for treatment; Adverse reactions related to treatment.	Preoperative chemo or chemoradio treatment.
Preoperative scores and scales	MUST score; HADS score; PSS score; EORTC-CR30/C29; LARS score (whenever possible).	
Radiological/endoscopic information	Date of colonoscopy; Colonoscopy findings; Date of MRI; MRI findings; Date of CT scan; CT scan findings; Preoperative evidence of distal metastasis; Metastasis sites; Preoperative evidence of synchronous CRC; Synchronous CRC localization; cTNM; Mesorectal fascia involvement; Date of restaging (if indicated); Type of restaging; cTNM of restaging.	Preoperative evidence of distal metastasis; Preoperative evidence of synchronous CRC; Synchronous CRC localization.
Surgical data	Date of surgery; Type of surgery; Surgery denomination; Start time; End time; Operative time; Surgery setting; Surgical approach; Type of anesthesia; Ileostomy/colostomy construction; Additional resections (e.g. hepatic resections); Surgical-related complications; EBL; Intraoperative transfusion; Drain placement.	Type of surgery; surgery denomination; operative time; surgery setting; surgical approach; Additional resections (e.g. hepatic resections).
Postoperative outcomes	Date of admission; Date of discharge; LOS; Discharge setting; 90-day postoperative complications; Clavien-Dindo classification (of the most severe complication); CCI (all complications); 48-h and 72-h postoperative pain (VAS scale); 24-h, 48-h, and 72-h CRP; Opioid medications; Time to first flatus; Time	LOS; 90-day postoperative complications; Clavien-Dindo classification (most severe complication); 90-day reoperation.

	to first bowel movement; Daily drain volumes (if applicable); 90-day reoperation; type of reoperation; 90-day readmission.	
**Pathological data**	pTNM classification; Tumoral stage (AJCC 8th edition); Grade of regression Dworak (if applicable); Number of harvested lymph nodes; Number of positive lymph nodes; Lymph nodes ratio; Circumferential margin positivity; Distance of the tumor from the distal margin; Resection margins; Mucinous phenotype; Signet-ring cells phenotype; Microsatellite status; Grade of differentiation; Extramural invasion; Perineural invasion; Lymphovascular invasion; Mutations test; mutated gene; type of mutation.	pTNM classification; Tumoral stage (AJCC 8th edition); Grade of regression Dworak (if applicable); Number of harvested lymph nodes; Number of positive lymph nodes; Lymph nodes ratio; Circumferential margin positivity; Distance of the tumor from the distal margin; Resection margins; Mucinous phenotype; Signet-ring cells phenotype; Microsatellite status; Grade of differentiation; Extramural invasion; Perineural invasion; Lymphovascular invasion; Mutations test.
**Postoperative scores and scales**	30-day: MUST score; HADS score; PSS score; EORTC-CR30/C29; LARS score (whenever possible). 6-month: MUST score; HADS score; PSS score; EORTC-CR30/C29; LARS score (whenever possible). 12-month: MUST score; HADS score; PSS score; EORTC-CR30/C29; LARS score (whenever possible).	
**Postoperative treatment information**	Adjuvant therapy; Type of adjuvant therapy; Start date; End date; Therapy duration; Hospital of therapy; Adverse reactions related to therapy; Withdrawal; Reason for withdrawal; Stoma closure (if applicable); Date of stoma closure.	Adjuvant therapy.
**Oncological outcomes**	Date of last follow-up; Status at last follow-up (alive/dead); Date of death; Cause of death; Cancer-related death; Time from surgery to death; Recurrence; Progression; Site of recurrence/progression; Date of recurrence or progression; Time from surgery to recurrence/progression; Time from surgery to last follow-up; Therapy for recurrence/progression; Type of therapy; Surgery for recurrence/progression; Type of surgery; Date of surgery for recurrence; Metachronous CRC tumors; Date of metachronous CRC	Status at last follow-up (alive/dead); Cancer-related death; Time from surgery to death; Recurrence; Progression; Time from surgery to recurrence/progression; Time from surgery to last follow-up; Metachronous CRC tumors; Other neoplasms.

	tumor diagnosis; Other neoplasms; Date of other neoplasm diagnosis.	

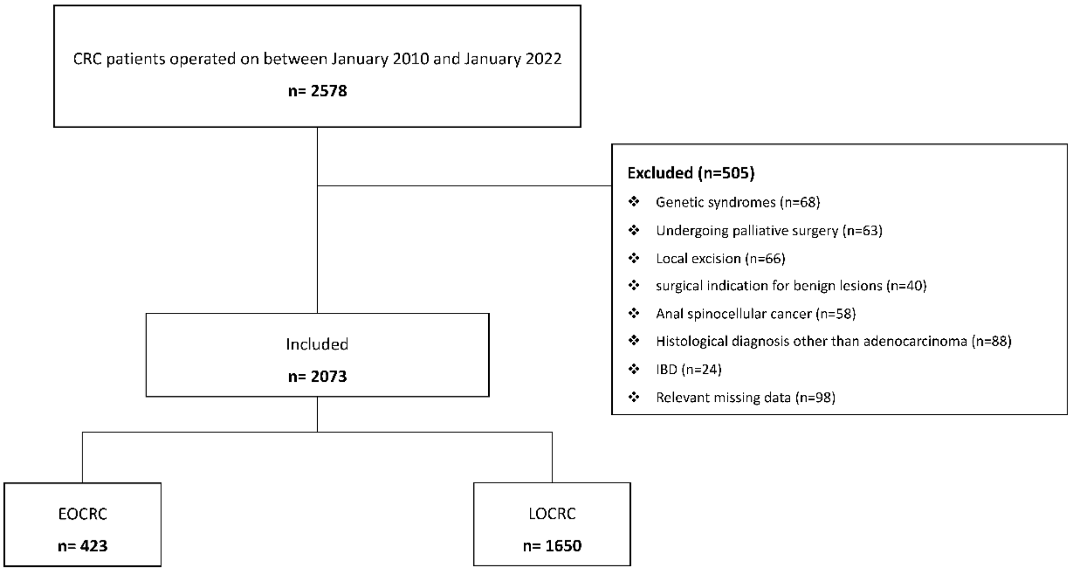

Figure S1: study inclusion flowchart.

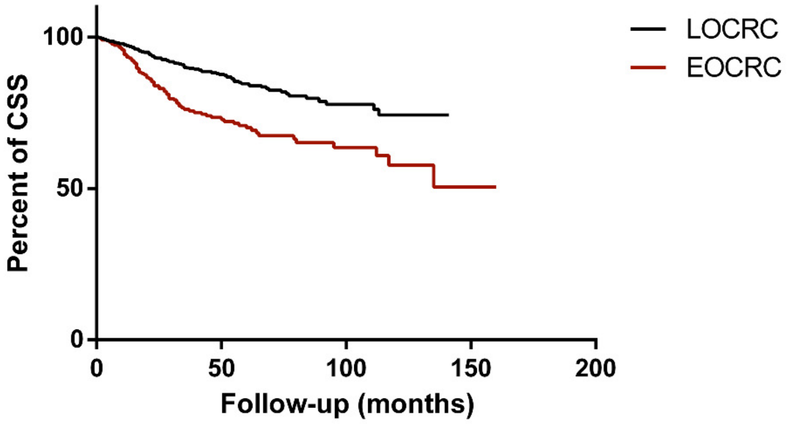

Figure S2: CSS (Cancer specific-free survival) of EORC (Early-onset colorectal cancer) (red line) and LORC (Late-onset colorectal cancer) (black line) patients. Data were compared with Kaplan-Meier analysis and Log-rank (Mantel-Cox) test (HR=2.09; 95% CI: 1.77-3.46; p< 0.0001). The CSS proportion in EOCRC patients at 36 months was 76% versus 89% in the LOCRC group (150/344 versus 501/1636 subjects at risk).
